# Correction of MHS Viscosimetric Constants upon Numerical Simulation of Temperature Induced Degradation Kinetic of Chitosan Solutions

**DOI:** 10.3390/polym8060210

**Published:** 2016-05-31

**Authors:** Vincenzo Maria De Benedictis, Giulia Soloperto, Christian Demitri

**Affiliations:** 1 DHITECH Scarl, Via Trinchese 61, Lecce 73100, Italy; vincenzomaria.debenedictis@unisalento.it (V.M.D.B.); giulia.soloperto@dhitech.it (G.S.); 2 Department of Engineering for Innovation, University of Salento, Via Monteroni, km 1, Lecce 73100, Italy

**Keywords:** chitosan, kinetic, degradation, acidic environment, numerical simulation

## Abstract

The Mark–Houwink–Sakurada (MHS) equation allows for estimation of rheological properties, if the molecular weight is known along with good understanding of the polymer conformation. The intrinsic viscosity of a polymer solution is related to the polymer molecular weight according to the MHS equation, where the value of the constants is related to the specific solvent and its concentration. However, MHS constants do not account for other characteristics of the polymeric solutions, *i.e.*, Deacetilation Degree (DD) when the solute is chitosan. In this paper, the degradation of chitosan in different acidic environments by thermal treatment is addressed. In particular, two different solutions are investigated (used as solvent acetic or hydrochloric acid) with different concentrations used for the preparation of chitosan solutions. The samples were treated at different temperatures (4, 30, and 80 °C) and time points (3, 6 and 24 h). Rheological, Gel Permeation Chromatography (GPC), Fourier Transform Infrared Spectroscopy (FT-IR), Differential Scanning Calorimetry (DSC) and Thermal Gravimetric Analyses (TGA) were performed in order to assess the degradation rate of the polymer backbones. Measured values of molecular weight have been integrated in the simulation of the batch degradation of chitosan solutions for evaluating MHS coefficients to be compared with their corresponding experimental values. Evaluating the relationship between the different parameters used in the preparation of chitosan solutions (e.g., temperature, time, acid type and concentration), and their contribution to the degradation of chitosan backbone, it is important to have a mathematical frame that could account for phenomena involved in polymer degradation that go beyond the solvent-solute combination. Therefore, the goal of the present work is to propose an integration of MHS coefficients for chitosan solutions that contemplate a deacetylation degree for chitosan systems or a more general substitution degree for polymers in which viscosity depends not only on molecular weight and solvent combinations.

## 1. Introduction

The viscosity study of polymers has been of continuing interest, mainly due to its fast measurement and importance in the characterization of the intermolecular interaction, as it describes the measure of the resistance to flow of a material, mixture, or solution. The equation that is generally used to represent the relationship between the intrinsic viscosity [η] and the weight-average molecular mass, *M*_w_, is the Mark–Houwink–Sakurada (MHS) equation: $\left[\eta \right]=KM_{W}^{a}$, where the proportionality constant *K* is characteristic of the polymer and solvent and the exponential *a* is a function of the shape of the polymer coil in a solution [1]. Therefore, the MHS equation provides highly important information for determining molecular weight, whose direct measurements otherwise would be extremely complicated. On the other hand, MHS equations enable the exploitation of easier measurement of the viscosity. Additionally, mathematical modeling could enable deepening experimental findings, allowing the simulation of parametric variation of the experimental conditions and the improvement of rheological models. However, most numerical simulations tend to study alteration of these structures and often investigates degradation kinetics of polymer-based device rather than of the bulk material [2]

Among natural polymers, chitosan, a β-(1-4) linked 2-amino, 2-deoxy,-d-glucan (Figure 1A), is the only amino polysaccharide that is present in large amounts in nature. It is the deacetylated derivative of chitin, the most abundant natural polymer on earth after cellulose. This biopolymer is synthesized by an enormous number of living organisms [3], such as marine crustaceans, like shrimp and crabs, and by some fungi like Basidiomycetes, Ascomycetes, and Phycomycetes [4,5], in which it is a component of the cell wall and structural membrane of the mycelium, stalk and spores [6]. Although many types of synthetic polymer formulations are being industrially produced, natural polymers, especially polysaccharides, are growing in interest applied to a range of technological fields [7,8,9,10,11,12]. Chitosan is biodegradable and biocompatible [13,14], having bioadhesive [15] and osteogenic properties [16,17,18,19,20,21,22,23,24,25]. It is a linear polycationic polysaccharide soluble only in acidic medium (p*K*a 6.5). Chitosan has been intensely investigated for the development of novel materials and sorption systems due to the presence of free amino groups in its structure, which allow its chemical modification [11,26,27,28]. Additionally, chitosan has attracted much interest in the biomedical industry because of its excellent antimicrobial activity and wound-healing acceleration properties [29]. In addition, thanks to its possible use in the development of controlled release implant systems [30,31,32,33], it has been widely used in industries including those focused on wastewater treatment [34], food [35], and cosmetics [36].

The rheological behavior of chitosan solutions has been widely reported in the literature [37,38,39]. In fact, it was demonstrated that the viscosity of concentrated chitosan solutions increased by increasing chitosan concentration, and that shear thinning behavior is observed for polymer concentrations above 5.0 g/L. The independence and dependence of the zero shear viscosity on the ionic strength and pH of the media, respectively, was also demonstrated [40,41,42].

The non-Newtonian behavior of chitosan solutions, which increases with the degree of deacetylation (DD), has been attributed to the chains’ expanded structure and to the increase of entanglements [43]. A reduction in the dynamic viscosity of chitosan solutions in acetic acid environment during storage has also been reported, due to polymer degradation [44,45]. Moreover, several studies have addressed chitosan thermal decomposition and stability [13,46,47,48,49,50,51,52]. In fact, it has been demonstrated that the mechanism of degradation of chitosan is mainly hydrolytic depolymerization of chains due to the acidic environment, as reported for other polysaccharides [53]. The breaking of the glycosidic bond at a given temperature is strongly influenced by the pH value and, therefore, by acidic concentration and metal impurities, which act as catalysts in the breaking process of polymer chains [54,55].

This hydrolytic mechanism generally involves three steps: (1) the glycosidic oxygen atom becomes protonated (*i.e.*, less basic), giving the conjugate acid; this step is rapid and the acid will exist in its equilibrium concentration; (2) a unimolecular heterolysis of the conjugate acid with the formation of a non-reducing end-group and a carbonium-oxonium ion; this step is slow and rate-determining; (3) a rapid addition of water to the carbonium-oxonium ion, with the formation of a reducing end-group and a proton (Figure 1B).

Moreover, in the case of the presence of strong acids (e.g., nitric acid), a complete deamination occurs [13,45,46,47,48,49]. In these extreme conditions, the full degradation of the polymeric structure is a consequence of all the aforementioned mechanisms. Nonetheless, the MHS equation is able to include information regarding solely the solvent involved with no reference to the DD. Kasaai in 2007 [56] proposed using two equations to determine *a* and *K* for chitosan in any solvent–temperature system. In particular, for a given degree of acetylation, pH and ionic strength, a deviation of up to 29% and 71% of the *a* and *K* values, respectively, was calculated experimentally. Further modeling studies of the chitosan hydrogel sphere response account for pH-related swelling and deswelling in a buffer solution [57]. However, none of them apply to our case, since the chitosan is not jellified, but rather in the form of aqueous solution, and measurement of the degree of deacetylation of the chitosan in the solution is beyond the scope of this work. The MHS parameters are calculated at standardized temperatures and, in many cases, these are not useful because of the errors they carry, and it then becomes very difficult to calculate the molecular weight [58]. The constant *K* reportedly decreases with increasing temperature in range 25–45 °C, the same temperature range, while the *a* value evaluated for the system decreased with increasing temperature [59].

This work evaluates how different factors contribute to degrade a dilute aqueous solution of chitosan occurring during the preparation and storage of the polymer solution. Moreover, the present work also addresses the correlation of the degradation rate as a function of several parameters, such as temperature, acidic concentration, and typology of acid (acetic acid *vs.* hydrochloric acid). Therefore, this numerical study aims at approaching the identification of the appropriate correction factor to the MHS coefficient in order to account also for DD.

The chain degradation was assessed through rheological measurements, Gel Permeation Chromatography (GPC), FT-IR spectra acquisitions, and Differential Scanning Calorimetry (DSC) on the sample. The final goal of the work is to understand the effect of the aforementioned parameters on the degradation rate, with the aim to control the final properties of the material. The outcomes of the work will be beneficial in order to increase the reproducibility during the characterization of chitosan-based devices.

## 2. Materials and Methods

### 2.1. Materials

Chitosan with medium molecular weight (MMW), glacial acetic acid (AA), hydrochloric acid 37% (HA) were purchased from Sigma Aldrich (Milan, Italy) and used without further purification. All solutions were prepared using water from a reverse osmosis purification system (General Waters Srl, Bologna, Italy) and filtered through a 0.45 mm Milipore syringe filter (Sigma Aldrich, Milan, Italy). The samples were prepared by suspending 2 g of chitosan in 100 g of AA (or HA) solutions at different concentrations in glass flasks. In Table 1, the complete list of the adopted sample codes and their concentrations are reported.

The flasks were immersed in a thermostatic bath at 25 °C and the suspension stirred gently for two hours with a mechanical stirrer. Each of the resulting solutions was divided in three different samples, and the subsequent thermal treatment was started immediately.

### 2.2. Rheological Characterization

After the complete dissolution, the samples were treated by immersion in a thermostatic bath at different temperatures (4, 30, and 80 °C). The degradation process was monitored continuously by measuring the dynamic viscosity of different samples taken from the same starting solution during the hydrolysis process at different time instants (*i.e.*, 0, 3, 24 h). The rheological characterization was carried out in a strain controlled Rheometer (Ares Rheometric Scientific, TA Instruments—Waters LLC, New Castle, DE, USA)

The tests were performed with a plate and plate flow geometry (radius = 12.5 mm) in a steady state mode at room temperature (25 °C), setting a shear rate ranging from 0.1 to 100 s^−1^. All experiments were carried out in triplicate, and the average and standard deviation values were evaluated.

### 2.3. Thermo Gravimetrical (TG) and Differential Scanning Calorimeter (DSC)

The thermal stability of control sample and chitosan films was measured through thermogravimetric analysis (TGA/DSC1 Star and System, METTLER Toledo, Columbus, OH, USA). To this aim, the samples were heated from 25 up to 800 °C at 10 °C/min in nitrogen atmosphere. A differential scanning calorimeter (DSC, Star and System, METTLER Toledo, Columbus, OH, USA) was used for DSC analyses. Samples were obtained by casting chitosan solutions after the thermal degradation treatment at different time instants (0, 3 and 24 h) into polystyrene petri dishes for film formation, and then dried in the oven at 30 °C for 48 h. The obtained films were peeled off gently and were further dried by keeping them in a desiccator (with silica gel desiccant, under vacuum) and tested without any further modification. An empty pan was used as a reference. For this purpose, the samples were heated from 25 °C up to 400 °C at 10 °C/min in nitrogen atmosphere.

### 2.4. Fourier Transformed Infrared Spectroscopy (FT-IR)

The Fourier transform infrared (FT-IR/6300 Jasco, Easton, MD, USA) spectrometer was used to monitor the degradation profile of the samples. These analyses were carried out on the same samples that had been prepared for the TG/DSC analyses, and used directly without any modification at a resolution of 4 cm^−1^, by 64 scans, at transmittance range from 4000 to 400 cm^−1^.

### 2.5. Gel Permeation Chromatography (GPC)

The molecular weight of chitosan in acetic acid solutions on samples after 24 h for each temperature was determined by gel permeation chromatography (GPC) with an Agilent 1100 HPLC Pump (Agilent Technologies, Santa Clara, CA, USA) and an Agilent 1200 Refractive Index Detector (Agilent Technologies, Santa Clara, CA, USA). The column used was PL-aquagel-OH Mixed-H (8 μm, 300 × 7.5 mm^2^) (Agilent Technologies, Santa Clara, CA, USA) and the eluent used was a buffer solution at pH 4.6 (0.5 M CH3COOH/0.5 M CH3COONa) with a flow rate 1 mL/min. The equipment was calibrated using Pullulan Standard Set (Sigma Aldrich, Milan, Italy). Results show the elution volumes of each sample, which are inversely proportional to the molecular weight.

### 2.6. Determination of the Degradation Rate and Activation Energy

Tanford [60] developed a simple equation able to describe a random depolymerization in case of a a single-stranded polymer according the following relationship:

(1) $$\frac{1}{\bar{DP_{n,t}}}=\frac{1}{\bar{DP_{n,0}}}+Kt,$$

where $\bar{DP_{n,t}}$ and $\bar{DP_{n,0}}$ are number of average degrees of polymerization, at times *t* and 0, respectively, and *k* is the rate constant for bond cleavage. The previous equation indicates that the inverse of molecular weight should increase linearly with the depolymerization time, since the $\bar{DP_{n}}$ values are proportional to molecular weight as follows:

(2) $$\frac{1}{\bar{M_{n}}}-\frac{1}{\bar{M_{n,0}}}=\frac{kt}{M_{0}},$$

where $\bar{M_{n}}=M_{0}\bar{DP_{n}}$. This means that the decrease in molecular weight is defined by the rate constant K.

The intrinsic viscosity [η] was calculated using the single-point determinations, like that based on the Solomon–Ciuta equation [51,61], as alternatives to dilution procedures by Huggins and Kraemer determinations:

(3) $$\left[\eta \right]=\frac{{\left[2\left(\eta_{\text{sp}}-\ln\eta_{r}\right)\right]}^{1/2}}{c}=\frac{{\left[2\left(\eta_{r}-1-\ln\eta_{r}\right)\right]}^{1/2}}{c}$$

where η_sp_ is the specific viscosity, η_r_ is the relative viscosity and *c* the concentration. Combining the MHS Equation (Mark–Houwink–Sakurada)

(4) $$\left[\eta \right]=K{\bar{M}}^{a},$$

with the previous equation leads to the following expression:

(5) $$\frac{1}{{\left[\eta \right]}^{1/a}}-\frac{1}{{\left[\eta \right]}_{0}{}^{1/a}}=k^{\prime }t,$$

where [η] and are [η]_0_ are the intrinsic viscosities at time *t* and time 0, *k′* = *k*/(2*M*_0_*K*^1/a^), *k* the rate of bond cleavage, *M*_0_ the monomer weight, and *K* and *a* are the constants of the MHS equation.

*K* and *a* for chitosan can be estimated as described in literature [62]. The rate constant *k′* for degradation is simply obtained by plotting $\frac{1}{{\left[\eta \right]}^{1/a}}$ *vs.* degradation time, and converted to *k* through the equation *k′* = *k*/(2*M*_0_*K*^1/a^). The degradation rate (*k*) can be used to find the activation energy by taking the natural logarithm of the Arrhenius equation:

(6) $$\ln k=\ln A-E_{a}/RT,$$

where *E*_a_ is the activation energy, *R* is the gas constant (8314 JK^−1^mol^−1^) and *T* is the absolute temperature. A plot of *ln k vs.* *1/T* gives a straight line whose slope is equal to −*E*_a_*/R*.

### 2.7. Numerical Simulations

A constant temperature of 80 °C (corresponding to 373.15 K) was assigned to each simulation. For all simulations, a depolymerization reaction was mimicked in which a polymer tends to convert into a monomer. Initial concentrations of the two species are 2% and 0% of the batch volume, respectively. The initial value of average molecular weight of chitosan fragments and the forward rate constant as obtained from Equation (6) were fed to each simulation.

Governing equations are the following:

(7) $$\frac{d\left(c_{i}V_{r}\right)}{d\left(t\right)}=V_{r}R_{i},$$

(8) $$V_{r}\sum_{i}c_{i}C_{p,i}\frac{dT}{dt}=Q+Q_{\text{ext}},$$

(9) $$Q=-V_{r}\sum_{j}H_{j}r_{i},$$

where Equation (7) represents species mass balance (*V* = reactor volume, *c* = species molar concentration, *R* = species rate expression), Equation (8) is the energy balance (*C* = molar heat capacity, *T* = temperature; *Q* = heat due to the chemical reaction; *Q*_ext_ = heat provided to the system) and Equation (9) is the heat of the reaction (*H* = enthalpy of the reaction; *r* = reaction rate, automatically calculated based upon species concentration and *k*). Thermal exchange of the reaction is assumed negligible (*Q*_ext_ = 0), as the flasks were immersed in a constant temperature bath. The simulation was conducted for a simulated time of 24 h (86,400 s) with a time interval of 1 s and the Backward Differentiation Formula (BDF) was used as time-stepping method.

Obtained values of varying concentrations could be employed to derive molecular weight variation according to the following expression:

(10) $$M=\frac{c_{p}M_{0h}+c_{m}M_{24h}}{c_{p}+c_{m}}\frac{1}{M_{0h}},$$

where *c*_p_ and *c*_m_ are, respectively, the concentration of the polymer and monomer, as numerically simulated, and *M*_0h_ and *M*_24h_ are the molecular mass values of the polymer obtained experimentally when the solvent is added to chitosan and after 24 h, by means of GPC analysis. The latter is usually set by providing, as well as standard fluid to compare the examined sample with, MHS coefficients. However, complex relationships between intrinsic viscosity and molecular weight are not limited to MHS, but, as earlier introduced, include parameters such as DD. Measured intrinsic viscosity and molecular weight, calculated as per Expression (10), could be inserted into Equation (4), in turn allowing calculation of MHS coefficient for each experimental condition at the highest temperature value, *i.e.*, 80 °C.

In particular, the intrinsic viscosity values for the four samples, determined experimentally at discrete time intervals throughout the duration of the experiment, were linearly interpolated to obtain intrinsic viscosity value for each simulated time instant and each calculated value of molecular weight. *K* and *a* were solved iteratively.

### 2.8. Statistical Analysis

All quantitative experiments were performed in triplicate and the results were expressed as mean ± standard deviation for *n* = 3. The statistical analysis of the data was conducted using one-way ANOVA. Differences between the groups with *p* < 0.05 were considered to be statistically significant.

## 3. Results and Discussion

In this study, the shear rate-dependent viscosity of chitosan solutions as a function of degradation time was evaluated.

Figure 2 shows the viscosity values (recorded at a shear rate of 1 s^−1^) at different time instants. It is evident that all the samples stored at lower temperature (4 °C) do not show any significant difference even after storage for 24 h. It is important to remark that this consideration remains valid also by varying the acidic concentration in both acetic and hydrochloric acid. In the case of the samples stored at 30 °C, there is a slight decrease in the viscosity in a range that could be considered significantly different only after 24 h. In the case of the samples stored in the presence of acetic acid, there is no significant difference between the 4 and 30 °C samples. Conversely, for the samples treated at higher temperatures, there is a dramatic decrease of the viscosity after 24 h. In this last case, the combination of the acidic environment and temperature plays an important role in the degradation profile of the polymer [63]. Similarly to what was observed for the samples with the higher concentration of hydrochloric acid, also in this case, the effect of acidic concentration is evident. In addition to this, non-Newtonian behavior is observed for all the samples. However, increasing the temperature leads to the appearance of Newtonian behavior: in fact, the decrease in the viscosity values was more pronounced for the samples treated at higher temperature *i.e.*, lower shear thinning and higher viscosities are observed at lower temperature. The obtained results show good agreement with previous studies [64,65,66].

In Figure 3, the plot of the viscosities of the different solutions stored for different time points at 4 °C is reported. It is important to note that the viscosity values are different between the tested samples. This is due to the difference in the ion strength of the solution, which increases by increasing the acidic concentration. The difference between the two acids is evident by analyzing the different ranges of viscosities. In particular, the solutions with AA show viscosity values one fold lower than those prepared with HA. This behavior is mainly due to differences in pH and ion strength of the solution. Higher pH combined with lower ion strength values interact with the polymer backbone by reducing the amine protonation on one hand but increasing the solubility of the polymer on the other hand. In such a condition, the viscosity of the solution decreases. Therefore, an excess of acetic acid, or other weak acid, is more favorable to chitosan solubility [7]. An opposite behavior affects the solubility of the samples with HA by increasing the viscosity of the solutions. In that case, the salting out occurs for HA concentrations larger than 1 M. In fact, only the dissociated fraction of the acid plays a role on the ionic strength of the solution and, even in presence of an excess of acetic acid, the degree of dissociation remains relatively low. No salting out was observed on the addition of concentrated acetic acid [41].

Figure 4 and Figure 5 are the TG and DSC curves, which characterize the thermal degradation of pure and degraded chitosan in nitrogen atmosphere. The TG curves were used to determine the mass decrement, while the DSC curves were used to evaluate the temperature of the starting point of decomposition (*T*_0_), taking as initial reaction the point at which the TG curve begins to deviate from its baseline. The temperature of the maximum speed of the process (*T*_m_) was determined from the maximum on the DSC curve (*i.e.*, DTG curves). The characteristic TG and DSC curves for the thermal degradation of pure chitosan are presented in Figure 4A. The thermogram exhibits two peaks that indicate two different phenomena. The first is due to the evaporation of the water absorbed by the chitosan, and from the TG curve it is possible to estimate that the weight loss due to this stage is approximately 9%. The second peak is due to water bound to chitosan, and to the liberation of small molecular products caused by chitosan thermal degradation [52]. This reaction is associated with a small endothermic reaction starting at about 200 °C, and a strong exothermic reaction starting at about 250 °C. The total weight loss in this second stage is approximately 45%. For comparison, the TG and DSC curves, which characterized the behavior of degraded (24 h, 80 °C) the CS-1M-HA sample in nitrogen atmosphere, are presented in Figure 4B. In that case, the DSC curve exhibits three peaks. The first one is related to the evaporation of water absorbed by chitosan as aforementioned; however, in this case, the amount of absorbed water is approximately 17%. The second and the third peaks correspond to the two-stage depolymerization of small molecular products, coupled with the thermal degradation of the sample. The second and the third peaks on the DSC curve occur at 220 and 250 °C, respectively, and they are accompanied by a significant mass decrement of approximately 45%. The lower degradation temperature is related with the fact that the material was already degraded during the thermal treatment. The presence of the second and the third endothermic peaks is associated with the presence of a small fraction of molecules with lower molecular weight that undergo evaporation at different temperatures [52]. The third peak is partially masked by a strong exothermic reaction due to complete polymer degradation.

To point out the differences in the thermal stability of chitosan over the degradation time between the two acids (AA and HA), the DSC curves of the samples during the degradation process at 80 °C (0, 3, 24 h) were acquired: results are reported in Figure 5. It is evident that the peak associated with the evaporation of the absorbed water is slightly affected by the degradation time but not by the nature of the acid. This is determined by the shift of the peaks toward lower temperature in comparison with the control. Conversely, there is a strong difference between the samples treated in AA or HA environments. In particular, for the samples presented in Figure 5C,D, which refer to the samples treated with HA, it is evident that a higher acidic concentration leads to the formation of a great number of molecules with a lower molecular weight. In fact, the endothermic peaks associated with the evaporation of these molecules are much more intense than the corresponding ones showed in Figure 5C. Furthermore, the obtained results confirm that HA affects the chitosan degradation temperature, which decreases from about 270 °C (for the samples treated with AA) to approximately 220 °C (in the case of CS-0.1M-HA). Moreover, the sample CS-1M-HA at 80 °C after 24 h does not show any evidence of exothermic peak as a consequence of a complete depolymerization mechanism.

The FT-IR spectra of pure chitosan (*t* = 0) and the samples treated for 3 and 24 h at 80 °C are illustrated in Figure 6. The FT-IR spectra of pure chitosan show a broad O-H stretching absorption band between 3400–3300 cm^−1^, methyl C-H stretching absorption 2870 and 2930 cm^−1^, methyl C-H bending absorption 1412 and 1380 cm^−1^, the polysaccharide structure between 1155–1032cm^−1^. Another major absorption band with 1560 cm^−1^ peak in the case of acetic acid and 1525 cm^−1^ peak in the case of hydrochloric acid represents the free primary amino group (–NH_2_) at C2 position of glucosamine. The peaks at 1648 and 1317 cm^−1^ are amide I and amide II, which indicate that the chitosan used in this investigation is not fully deacetylated.

The obtained spectra suggest that chitosan and depolymerized chitosan are very similar. This, in turn, demonstrates that the process of depolymerization caused no chemical change in the structure of the polymer except reduction in molecular weight, which is evident from the change in viscosity [49]. This confirms the mechanisms presented in Figure 1B. In fact, there is no evidence of any deamination showed by the intense reduction of 1560 cm^−1^ peak of free primary amino group, different from what has been reported for other strong acids (e.g., nitric acid) [45,48].

Change in molecular weight of chitosan solution subjected to different thermal treatments in AA was also monitored by gel permeation chromatography (Figure 7).

Also in this case, a similar trend of molecular weight decrease with increase of the storage at higher temperature can be observed. Shortening in sigma of Gaussian peaks with temperature (molecular weight decrease) also indicates narrowing of molecular weight distribution to reach values of 460 and 453 kDa for CS-0.1M-AA and CS-1M-AA, respectively (Table 2).

The first order rate constant (*k*) of glycosidic bond cleavage can be used to determine the activation energy (*E*_a_) of depolymerization using the Arrhenius equation (Equation (6)). The results are reported in Table 3, the means for each group show a difference statistically significant with *p* < 0.005.

The obtained values were very low if compared with those reported in literature [53,67,68], which refer to thermal degradation of chitosan chloride (109–114 kJ/mol), and other glycosidic compounds such as methyl-2-acetamido-2-deoxy-13-d-glucopyranose (119 kJ/mol) and methyl-2-amino-2-deoxy-13-d-glucopyranose (151 kJ/mol). In fact, these values were obtained by aging the solid powder at different temperatures (from 60 up to 120 °C) and time points. In the experiments performed in this work, the obtained values of *E*_a_ are compared with the thermal degradation of chitosan dissolved in acetate buffer 0.2 M (15–80 kJ/mol) [51]. These different values are due to a difference between acid hydrolysis of chitosan in solution and in the solid state. In solution, the thermal mobility of polymer chains and solvatation effects of protons promote hydrolysis. However, these effects are not evident in the solid state due to both a loss of free water at higher temperatures and lower chain mobility.

It can be seen that, for the full set of chitosan solutions (Table 3), the degradation rate constant is lower at 30 °C and higher at 80 °C. Furthermore, the degradation rate constant ratio (DRCR = k80 °C/k30 °C), indicating the sensitivity of the solutions to temperature variations, is reduced for diluted acid solutions and increased for concentrated acid solutions for both acid types. In particular, the DRCR value for concentrated hydrochloric acid is 5.5 times higher than the corresponding result obtained in a concentrated acetic acid environment (672 *vs*. 124), and 51.7 times in diluted hydrochloric acid (672 *vs*. 13). This difference indicates that the degradation rate of chitosan in concentrated hydrochloric acid solutions is very sensitive to temperature variations. Moreover, it was observed that even if the activation energies are similar between the tested samples, consistent with decreased depolymerization at lower pH values [69], the differences in the corresponding degradation rate values could be attributed to the different solubility of chitosan in this kind of system (*i.e.*, solutions with high ionic strength).

Numerical calculations of polymer degradation allow for obtaining variations of molecular weight over the 24 h for any solvent employed. Specifically, molecular weight, normalized on its respective initial value, presented the largest variation for 80 °C temperature applied (Figure 8).

Molecular mass of polymers and resulting oligomers for CS-1M-HA have been assigned arbitrarily, as GPC based measurements were not reliable due to the extreme depolymerization of chitosan. On the other hand, simulated values of polymer and so-called monomer concentrations were preliminary tested for independence from assigned molecular weight and resulted in being unaffected. Interestingly, it could be noted how acetic acid in a concentration ten-fold that of hydrochloric acid has comparable effects on chitosan degradation.

We observed that, applying MHS experimental coefficients [41] back to the GPC measured molecular weights, resulting values of intrinsic viscosity largely differ from the rheology. Such differences were not homeogeneous and calculated viscosities were not always larger than those measured, as reported in Table 4.

Table 5 reports values of calculated MHS coefficients as well as their experimental values, as available from the literature. The stronger acid at the highest concentration resulted in a noticeably high value of *K*.

Whereas the proportionality constant *K* is characteristic of the polymers and solvents and the exponential *a* is a function of the shape of the polymer coil in a solution, most calculated values of the exponential goes beyond the limits indicated by the literature [41]. Interestingly, MHS coefficients are within the same order of magnitude for the CS-0.1M-AA with the calculated value of *a* being more than two times larger than the experimental one; nonetheless, such a difference appears sufficient to lead to underestimating the value of intrinsic viscosity by about 50% when the experimental coefficients are used. CS-1.0M-AA and CS-0.1M-HA present a calculated value of *a* around four times (4.33 and 3.87, respectively) larger than the experimental yet, leading to small overestimation of the intrinsic viscosity using the experimental coefficients. For the last sample, characterized by a strong highly concentrated acid, the value of *a* is in the range from 0.0–0.5, reflecting a rigid sphere in an ideal solvent [42], which better represents the complete degradation occurring using 1M-HA as solvent. An extremely high value for *K* surely deserves further investigation, although the calculated value of *K* is lower than the experimental one for the other samples.

## 4. Conclusions

Prior to the preparation of chitosan-based materials, hydration and solubilization of chitosan suspensions at low pH are required. Heating, mixing and an acidic environment are important operations which are combined together to obtain a homogeneous starting solution. Several studies in the literature already indicate that these conditions promote polymer degradation; however, only limited information is available on the effect of the above-mentioned conditions on the subsequent final material properties. On such a basis, in this study the effect of mixing conditions, *i.e.*, time, temperature and acidic environment, on final gel characteristics, was investigated. Thermal treatment coupled with acidic environment was found to be sufficient to change chitosan molecular properties in different conditions, demonstrating that lower temperatures and a weak acid do not significantly affect the properties of the solution. Conversely, a higher temperature combined with longer exposure time dramatically affects the properties of chitosan in terms of reduction of rheological properties and molecular weights. The depolymerization rate constants (at 4, 30 and 80 °C) and activation energies for the chitosan solutions were estimated from intrinsic viscosity by rheological measurements. The relationships between molecular weight and intrinsic viscosity, as expressed by MHS constants, appeared to not be properly described, especially for chitosan solutions, since MHS constants do not take deacetylation degree into account. On the other hand, illustrated experimental tests provide fast tools to quantify chitosan depolymerization and may be used to determine the role of other factors such as acid type, acidic concentration, time and temperature on chitosan stability. The mechanism of degradation is a complex process and most likely involves two separate paths, such as the acidic environment generated by the solutions, resulting in polymer main chain scission and thermal degradation and hydrolysis induced by heat, that cause visible changes in the rheological properties without contributing significantly to the overall molecular weight degradation process. Finally, a comprehensive modeling of the rheological properties of polymer solution is required, mainly employing solutions with filtered molecular weight and a wider range of concentration values for the selected solvent, in order to find the appropriate mathematical frame to describe molecular weight variations in relationship to DD and intrinsic viscosity.

## Figures and Tables

**Figure 1:**
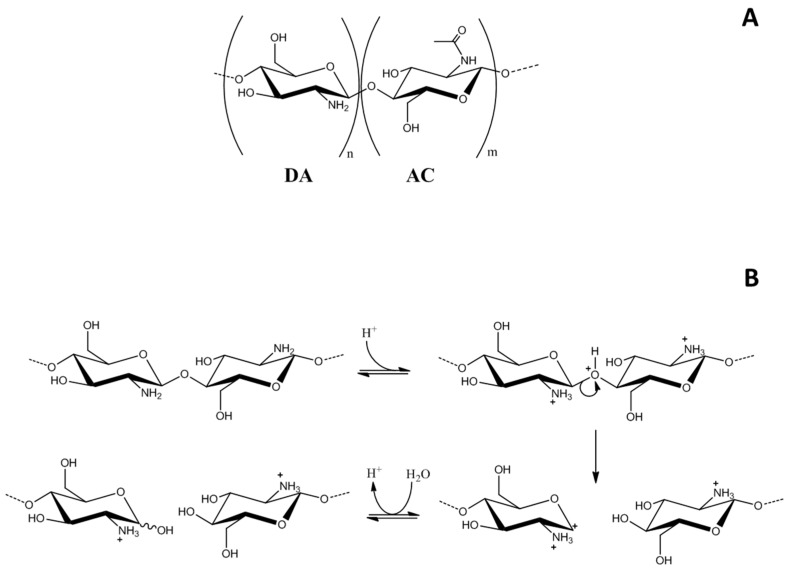
(**A**) Schematic representation of the structure repeat units of chitosan; (**B**) schematic representation of the chitosan degradation mechanisms.

**Figure 2:**
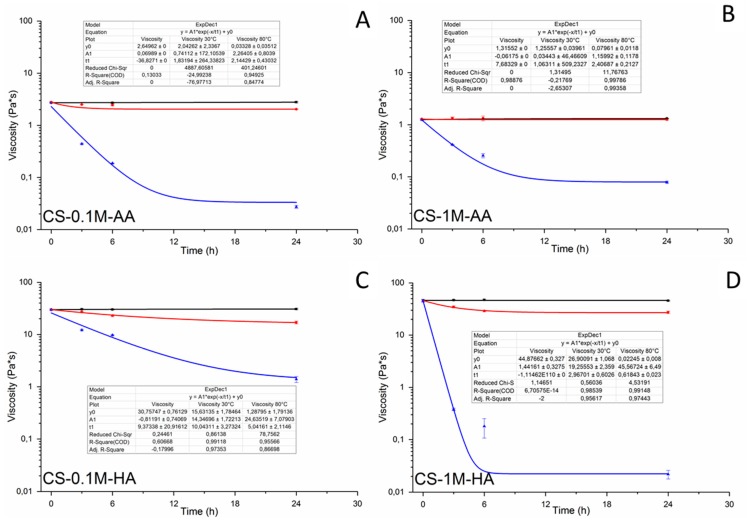
Viscosity of the different sample solutions stored for different time points at 4 °C (**square**), 30 °C (**circle**) and 80 °C (**triangle**). Please note that some symbols overlap the standard deviation bars. (**A**) CS-0.1M-AA; (**B**) CS-1M-AA; (**C**) CS-0.1M-HA ; (**D**) CS-1M-HA.

**Figure 3:**
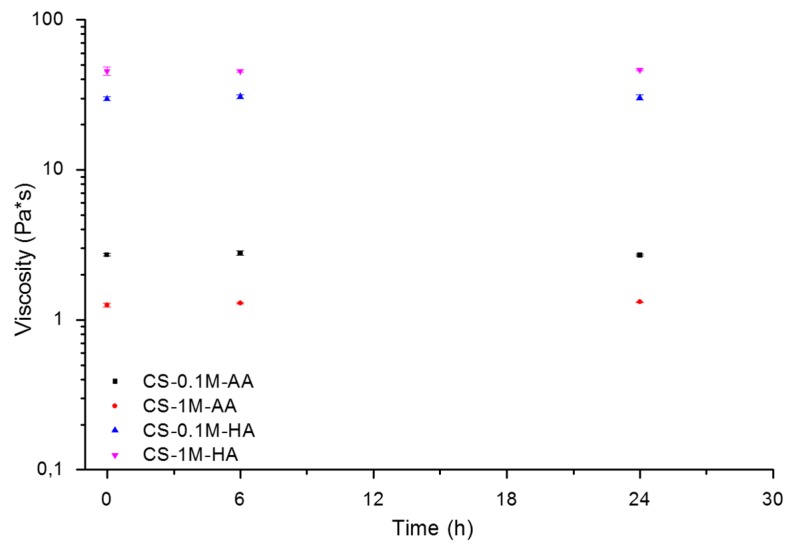
Viscosity of the different sample solutions stored for different time points at 4 °C. Please note that some symbols overlap the standard deviation bars.

**Figure 4:**
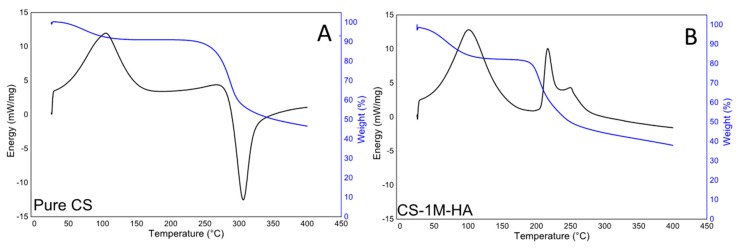
(**A**) TG and DSC curves for the thermal degradation of pure chitosan; (**B**) degraded (24 h, 80 °C) CS-1M-HA.

**Figure 5:**
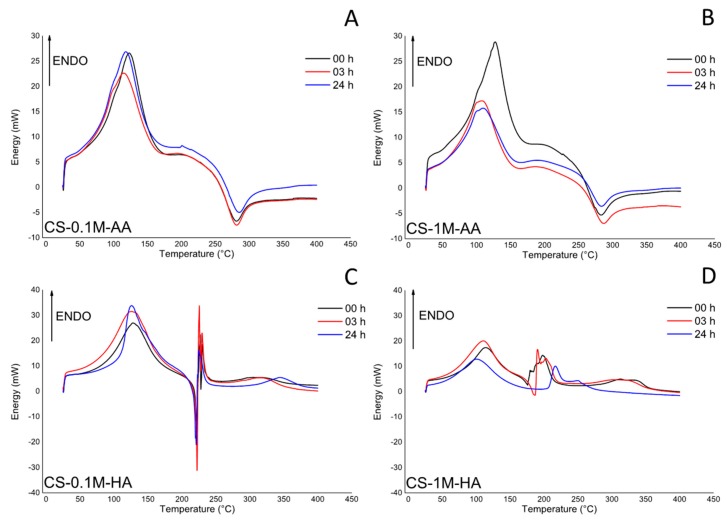
DSC thermograms of chitosan samples stored ad different time instants at 80 °C. (**A**) CS-0.1M-AA; (**B**) CS-1M-AA; (**C**) CS-0.1M-HA ; (**D**) CS-1M-HA.

**Figure 6:**
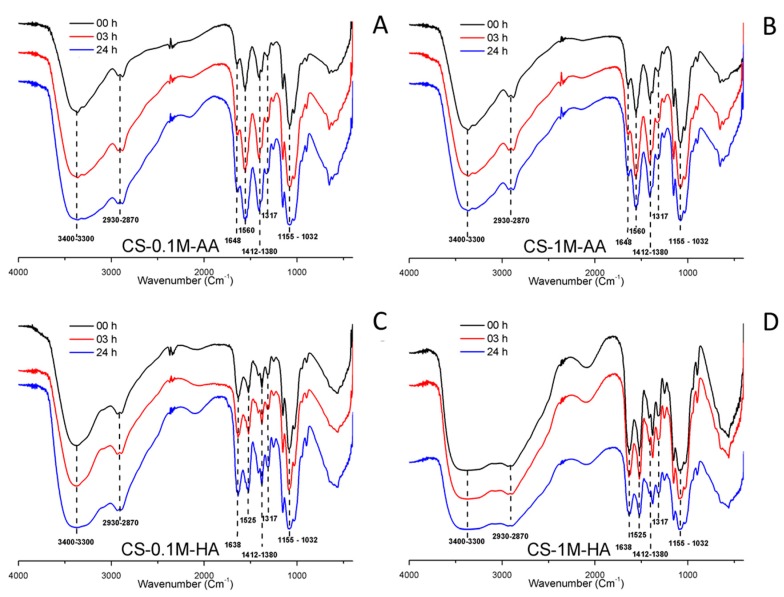
FT-IR spectra of chitosan samples stored at different time instants at 80 °C. (**A**) CS-0.1M-AA; (**B**) CS-1M-AA; (**C**) CS-0.1M-HA; (**D**) CS-1M-HA.

**Figure 7:**
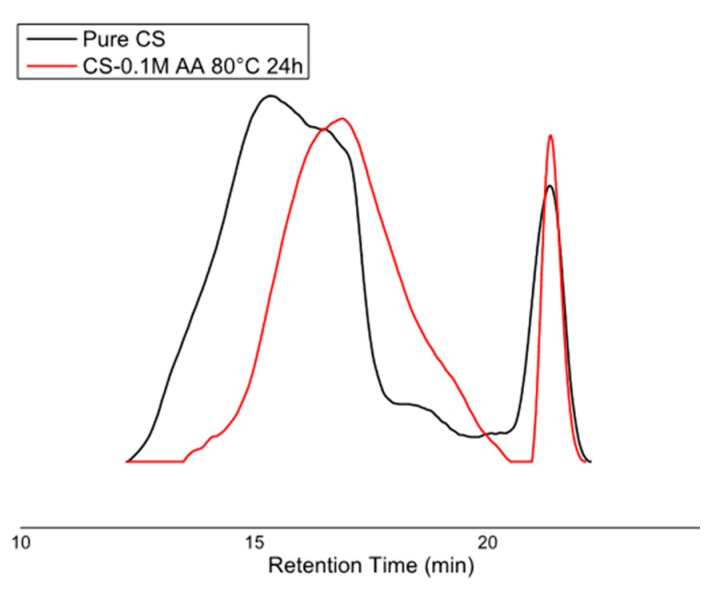
GPC elution profiles of pure chitosan and 2% *w/v* chitosan in 0.1 M acetic acid solution exposed to 80 °C for 24 h.

**Figure 8:**
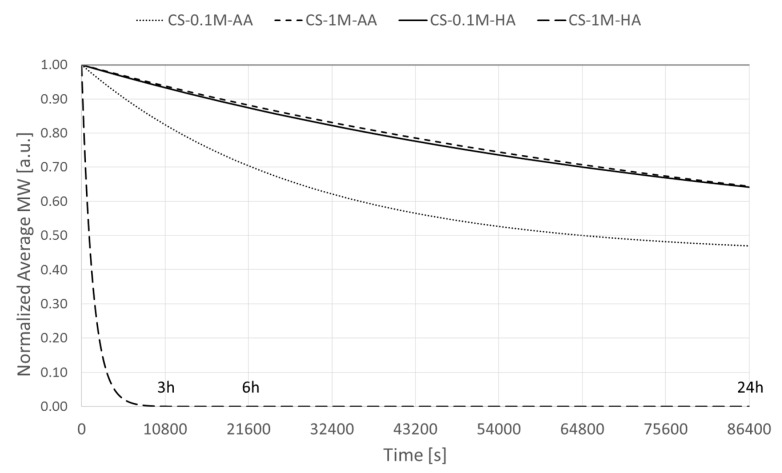
Normalized values of molecular weight over 24 h (86,400 s) time 2% chitosan solution with different solvent types and concentrations (please refer to Table 1 for sample coding) at 80 °C.

**Table 1:** Sample codes, compositions and pH of the resulting solutions.

Sample code	Chitosan	Acetic acid	Hydrochloric acid	pH
CS-0.1M-AA	2%	0.1 M	na	5.10
CS-1M-AA	2%	1.0 M	na	3.10
CS-0.1M-HA	2%	na	0.1 M	1.20
CS-1M-HA	2%	na	1.0 M	0.20

**Table 2:** Molecular weights, with respective standard deviations, for the indicated samples at initial and final time instants at 80 °C.

Sample code	Molecular weight [kDa] *t* = 0, *T* = 0 °C	Molecular weight [kDa] *t* = 24 h, *T* = 80 °C
CS-0.1M-AA	995.15 ± 4.03	824.0 ± 23.1
CS-1M-AA	1,003.00 ± 9.90	453.50 ± 67.17

**Table 3:** Degradation rate at 30 and 80 °C, Degradation rate ratio, and activation energy of solution compositions.

Sample code	Degradation rate constant k30 °C (h^−1^)	Degradation rate constant k80 °C (h^−1^)	Rate constant ratio k80 °C/k30 °C	Activation energy *E*_a_ (kJ/mol)
CS-0.1M-AA	0.0015 ± 0.0002	0.1261 ± 0.0008	84	72.325 ± 1.509
CS-1M-AA	0.00028 ± 0.00004	0.0348 ± 0.0025	124	55.860 ± 7.416
CS-0.1M-HA	0.0032 ± 0.0280	0.0423 ± 0.0018	13	59.478 ± 3.073
CS-1M-HA	0.0038 ± 00012	2.5330 ± 0.1084	672	75.704 ± 9.786

**Table 4:** Percentage of variation of measured viscosity with respect to its values calculated by means of MHS, maximum and minimum absolute variations are highlighted.

Sample ID	Intrinsic viscosity difference (%)	
	*T* = 0 h	*T* = 80; *t* = 24 h
CS-0.1M-AA	−52.40	60.81
CS-1.0M-AA	13.92	51.65
CS-0.1M-HA	0.34	59.17
CS-1.0M-HA	1,802.28	–

**Table 5:** Experimental MHS coefficients according to Kasaai [56] and MHS coefficients calculated upon numerical simulations. Refer to Table 1 for sample coding.

Sample ID	Exp		Calc	
	*a*	*K*	*a*	*K*
CS-0.1M-AA	1.26	3.04 × 10^−5^	2.72	3.01 × 10^−5^
CS-1.0M-AA	0.85	0.0138	3.68	3.63 × 10^−8^
CS-0.1M-HA	0.78	0.175	3.02	7.04 × 10^−4^
CS-1.0M-HA	0.66	0.0585	0.16	2,624.68
